# Syringic Acid Alleviates Cesium-Induced Growth Defect in *Arabidopsis*

**DOI:** 10.3390/ijms21239116

**Published:** 2020-11-30

**Authors:** Eri Adams, Takae Miyazaki, Ju Yeon Moon, Yuji Sawada, Muneo Sato, Kiminori Toyooka, Masami Yokota Hirai, Ryoung Shin

**Affiliations:** RIKEN Center for Sustainable Resource Science, 1-7-22 Suehirocho, Tsurumi-ku, Yokohama, Kanagawa 230-0045, Japan; eri_adams@galdieria.com (E.A.); takae.miyazaki@riken.jp (T.M.); juyeon.moon@riken.jp (J.Y.M.); yuji.sawada@riken.jp (Y.S.); muneo.sato@riken.jp (M.S.); toyooka@riken.jp (K.T.); masami.hirai@riken.jp (M.Y.H.)

**Keywords:** cesium, lignin, REF4, syringic acid

## Abstract

Syringic acid, a phenolic compound, serves a variety of beneficial functions in cells. Syringic acid increases in plants in response to cesium, and exogenous application of syringic acid resulted in a significant attenuation of cesium-induced growth defects in *Arabidopsis*. In addition, cesium or syringic acid application to plants also resulted in increased lignin deposition in interfascicular fibers. To better understand the role of lignin and syringic acid in attenuating cesium-induced growth defects, two mutants for *Arabidopsis* *REDUCED EPIDERMAL FLUORESCENE 4 (REF4*) and fourteen *laccase* mutants, some of which have lower levels of lignin, were evaluated for their response to cesium. These mutants responded differently to cesium stress, compared to control plants, and the application of syringic acid alleviated cesium-induced growth defects in the *laccase* mutants but not in the *ref4* mutants. These findings imply that lignin plays a role in cesium signaling but the attenuation of cesium stress defects by syringic acid is mediated by regulatory components of lignin biosynthesis and not lignin biosynthesis itself. In contrast, syringic acid did not alleviate any low potassium-induced growth defects. Collectively, our findings provide the first established link between lignin and cesium stress via syringic acid in plants.

## 1. Introduction

Excessive levels of cesium in soils negatively impact plant growth mainly through a restriction in the ability of plants to take up potassium due to the chemical similarity between cesium and potassium [1,2,3,4,5,6,7,8]. The regulation of cesium uptake and plant responses to cesium stress, however, have not been thoroughly elucidated. We previously reported that cesium stress in plants induces jasmonic acid biosynthesis and signaling, and that jasmonic acid functions antagonistically in the induction of the expression of *HIGH AFFINITY K^+^ TRANSPORTER5* (*HAK5*) [1]. Cesium stress alters the level of amino acids in plants, including cysteine and phenylalanine, and cysteine is associated with cesium accumulation in plants [9].

Syringic acid, a natural phenolic compound, is found in most plant species and some fungal species [10,11]. Syringic acid comprises a single benzene ring with two methoxy moieties attached to the ring at positions 3 and 5, which play an important role in radical-scavenging activity, especially in conjunction with 2,2-diphenyl-1-picryl and β-carotene [12,13]. Application of syringic acid to plants increases antioxidant and peroxidation levels and can be used to alleviate oxidative stress [14]. Syringic acid protects plant, animal, and human cells against lipid and protein oxidation, inflammation, cardiotoxicity, protein carbonylation, osteoporosis, neuro damage, and oxidative stress, and also plays a preventive role against diabetes and ischemia reperfusion [13,15,16,17,18,19,20,21].

Syringic acid is synthesized through the shikimic acid pathway [22] and is derived from phenylalanine by β-oxidation [23,24]. Sinapyl alcohol serves as the precursor of syringyl lignin, which is a source of lignin-derived syringic acid [13,22,23,24]. Lignin, a component of plant secondary cell walls, is synthesized from aromatic compounds. Lignin provides the physical strength to the secondary cell wall of xylem conduits that enables long-distance water transport in plants and creates both a physical and chemical barrier against pathogens [25]. Alterations in lignin biosynthesis and/or deposition in plants often modify defense responses and plant growth [25]. The multicopper-containing enzymes, laccases, are expressed in lignifying tissues and are involved in lignin polymerization [26]. Syringic acid contributes to the architectural integrity of lignin [13] and is a target for laccases, a monolignol catalyzing enzyme [23]. Mutation of several lignin biosynthesis enzymes, such as cinnamyl alcohol dehydrogenase, laccases, and peroxidases, reduces lignin accumulation and growth in plants [25]. The regulatory mechanisms and crosstalk that exist between lignin and plant growth, however, are thought to be complex.

The plant hormone, jasmonic acid, was confirmed as an upstream component of both plant growth and lignin biosynthesis [27]. The transcriptional coregulatory mediator complex, MED5a (also called REDUCED EPIDERMAL FLUORESCENE 4, REF4, At2g48110) and MED5b (also called REF4-RELATED 1, RFR1, At3g23590), participates in the homeostatic repression of the phenylpropanoid pathway [28]. The physically interacting REF4 and RFR1 transcriptional coregulatory complex provides the bridge between the basic transcriptional machinery and the specific transcription factors, including various abiotic stress-related transcription factors and phenylpropanoid pathways [28,29,30]. *Arabidopsis* semidominant *ref4* mutants exhibit partial dwarfism and reduced levels of lignin and sinapolymalate [31]. MED complexes interact with jasmonic acid-activated transcription factors and contribute to plant immunity by balancing the activity of jasmonic acid and salicylic acid pathways [32,33].

In the present study, an increase in syringic acid levels and a greater deposition of lignin in *Arabidopsis* plants were observed in response to cesium. In order to elucidate how lignin is functionally involved in cesium stress, and its association to syringic acid, the mutants of a lignin polymerization-related enzyme, laccase, and mutants related to the regulation of lignin biosynthesis, *ref4*, were utilized for the analysis. Results indicate that lignin mediates cesium stress in plants via syringic acid.

## 2. Results

### 2.1. Increased Levels of Syringic Acid in Cesium-Treated Arabidopsis Shoots

**Figure 1:**
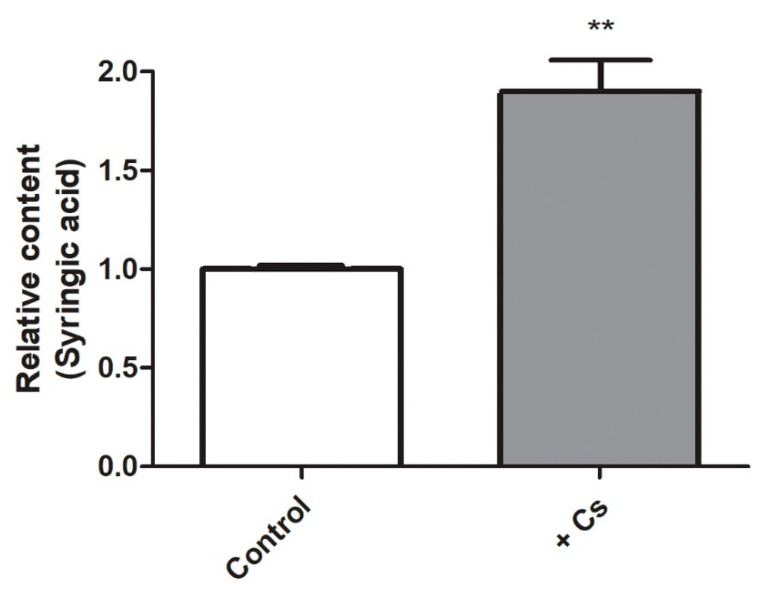
Relative levels of syringic acid in *Arabidopsis*. The levels of syringic acid were analyzed in shoots of cesium-treated *Arabidopsis* plants by liquid chromatography coupled with tandem quadrupole mass spectrometry (LC-QqQ-MS). Results are presented as a ratio between the level of syringic acid in untreated control plants versus the level in cesium-treated plants. The white bar indicates the level of syringic acid in shoots of control plants and the grey bar indicates the level of syringic acid in shoots of cesium-treated *Arabidopsis* plants. Error bars represent the standard error (SE). A *t*-test was conducted and ** indicates a significant difference between treated and untreated plants (*p* < 0.01, *n* = 4).

A previous metabolic profiling analysis of *Arabidopsis* wild-type Col-0 revealed a large metabolic shift in plants exposed to cesium [9]. The level of syringic acid was analyzed to determine if it was affected by cesium. *Arabidopsis* seedlings were grown for eight days with or without exposure to 0.3 mM cesium. Afterwards, shoots and roots were separately harvested and syringic acid levels were quantified using liquid chromatography coupled with tandem quadrupole mass spectrometry (LC-QqQ-MS) (Figure 1 and Figure S1). Results indicated that syringic acid levels were approximately doubled in shoots in response to the cesium treatment (Figure 1). In contrast, no alterations in syringic acid levels were observed in roots (Figure S1), indicating that syringic acid-related responses to cesium occur primarily in shoots of *Arabidopsis* rather than roots.

### 2.2. Syringic Acid Treatment Alleviates Cesium-Induced Growth Inhibition

**Figure 2:**
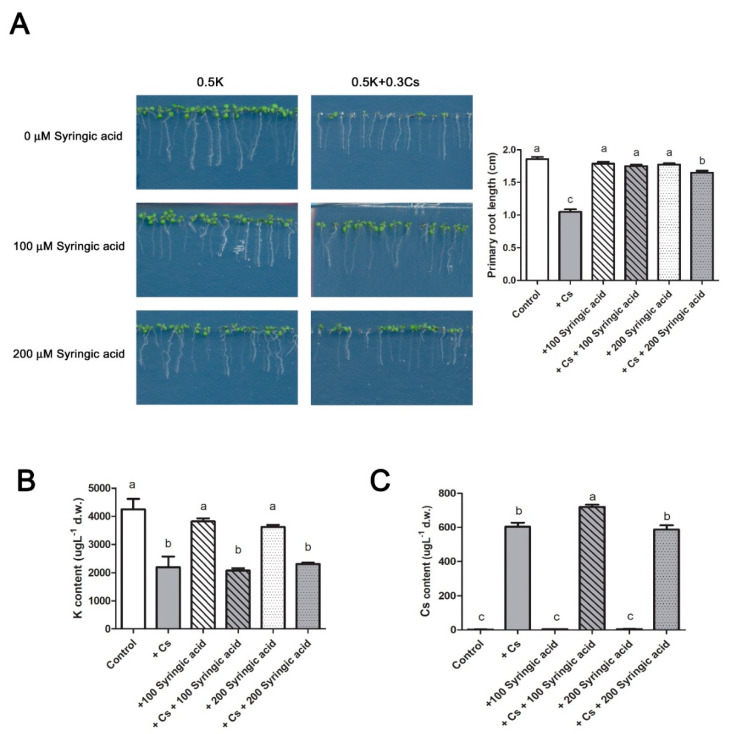
Application of syringic acid alleviates cesium-induced growth inhibition. (**A**) Wild-type *Arabidopsis* Col-0 was grown on media containing 0.5 mM potassium (K) and 0.3 mM cesium (Cs) supplemented with 0 μM, 100 μM, or 200 μM syringic acid for 8 days. Potassium (**B**) and cesium (**C**) levels were quantified for each treatment. Statistically significant differences were determined by a one-way ANOVA with Bonferroni’s multiple comparison post-test and indicated with different letters (*n* > 60 seedlings, *p* < 0.05). Error bars represent the SE.

Syringic acid was applied in conjunction with cesium to *Arabidopsis* in order to determine how syringic acid impacts the plant response to cesium (Figure 2). Results indicated that the cesium-induced growth inhibtion of plants was nearly completely alleviated by the syringic acid application (Figure 2A). Potassium and cesium levels were assessed using a flame atomic absorption spectrometer. The level of potassium was not altered by syringic acid treatment (Figure 2B), but the level of cesium was slightly increased by treatment with 100 μM syringic acid (Figure 2C).

Previous studies demonstrated that jasmonic acid is involved in cesium-induced root growth retardation [1,9]. In the present study, the root growth retardation by cesium was not found in *Arabidopsis* Col-0 plants that were concomitantly treated with 100 µM syringic acid and cesium. In contrast, there was mild retardation of root growth by a combined treatment with 200 µM syringic acid and cesium (Figure 2A). However, a clear alleviative effects of syringic acid was observed in shoots (Figure 2). Potassium starvation has been shown to induce an increase in ethylene and reactive oxygen species (ROS) that is produced by the activity of nicotinamide adenine dinucleotide phosphate (NADPH) oxidases [34,35]. In this regard, syringic acid is also known to provide a protective role against oxidative stress [36,37]. Therefore, based on these previous findings, NAPDH oxidase mutants [38,39] were evaluated for their response to cesium and to determine if their response was altered by the application of syringic acid (Figure S2). An ethylene insensitive mutant, *ein*2 (Figure S2A–C) [38], and an NADPH oxidase mutant, *respiratory burst oxidase homolog C* (*atrboh*-c) (Figure S2D–F), were found to be more tolerant to cesium stress. In comparison to control plants, most of the tested mutants showed a similar level of alleviation of cesium-induced growth defects in response to treatment with syringic acid (Figure S2). These results indicate that ethylene and ROS produced by NADPH oxidases were not likely to be involved in syringic acid-derived alleviation of cesium stress.

**Figure 3:**
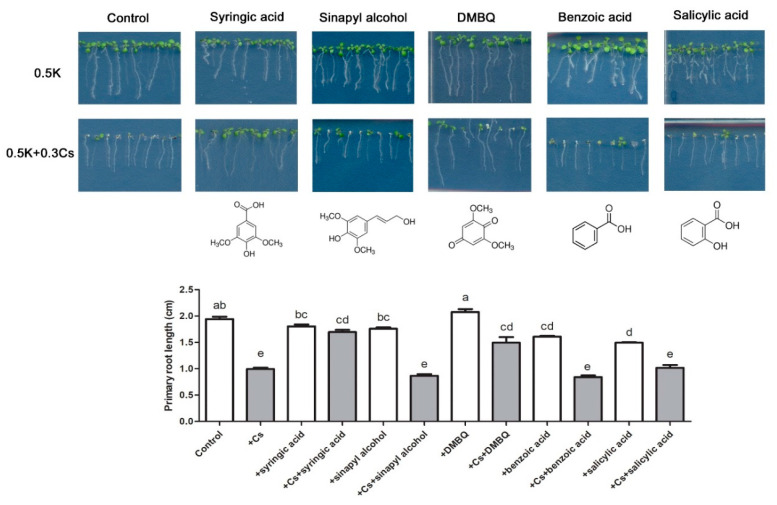
Phenotype analysis of cesium-induced growth inhibition in *Arabidopsis* seedlings treated with various phenolic compounds. Wild-type *Arabidopsis* Col-0 was grown for 8 days on media containing 0.5 mM potassium (K) and 0.3 mM cesium (Cs) supplemented with either 100 μM syringic acid, 100 μM sinapyl alcohol, 10 μM 1,6-dimethoxybenzoquinone (DMBQ), 10 μM benzoic acid, or 10 μM salicylic acid. Statistically significant differences were determined by a one-way ANOVA with Bonferroni’s multiple comparison post-test and are indicated with different letters (*p* < 0.05). Error bars represent the SE.

Since syringic acid is a phenolic compound composed of a single benzene ring, phenolic compounds with a similar structure were also evaluated to determine if they could alleviate cesium-induced growth retardation (Figure 3). Results indicated that the evaluated simple benzene ring compounds, benzoic acid and salicylic acid, were not able to completely alleviate growth retardation induced by treatment with 0.3 mM cesium [1]. Additionally, negative effects on root growth were observed by treatment with benzoic acid and salicylic acid, even without cesium treatment (Figure 3). One of the monolignols and a lignin precursor, sinapyl alcohol, did however provide a slight alleviation of the growth retardation induced by cesium. Treatment with 1,6-dimethoxybenzoquinone (DMBQ) resulted in a modest reduction in cesium-induced growth retardation. Specifically, growth retardation was 70% more alleviated in cesium-treated plants compared to controls, and approximately 30% less alleviated compared to syringic acid-treated plants (Figure 3). Notably, the DMBQ treatment improved root growth whether the plants were exposed to cesium or not. The degree of attenuation by DMBQ, however, was much smaller than by syringic acid treatment (Figure 3). Collectively, these data indicate that syringic acid is a phenolic compound that greatly alleviates cesium stress in *Arabidopsis*, but alleviation is not a general characteristic of phenolic compounds containing a single benzene ring.

### 2.3. Cesium and Syringic Acid Treatments Result in Increased Lignin Deposition in Arabidopsis

**Figure 4:**
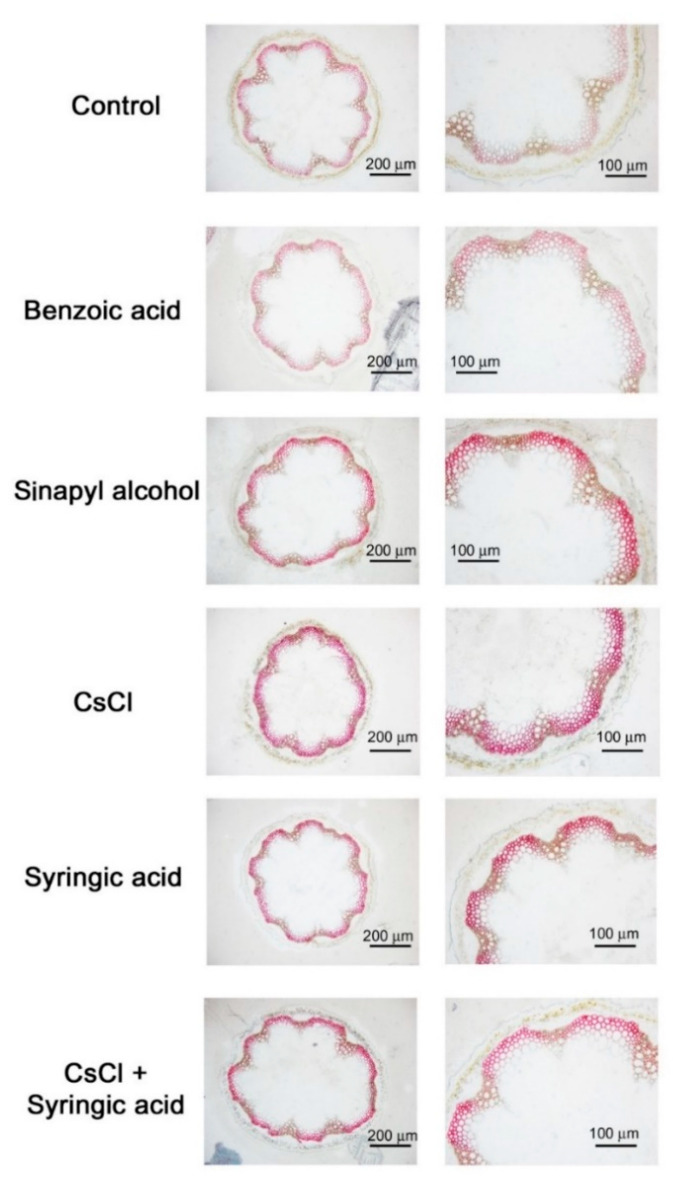
Histochemical analysis of lignin deposition in *Arabidopsis* stems. Soil-grown *Arabidopsis* plants were treated with Milli-Q water as a control, syringic acid, sinapyl alcohol, benzoic acid, CsCl, or a combined treatment of CsCl and syringic acid. The cryosectioned samples were 20 µm thick and stained for syringyl lignin. Images were analyzed and digitally photographed under an optical microscope.

The degradation of lignin is known to release phenolic compounds, including syringic acid, and syringic acid is also known to provide structural integrity to lignin [13,40]. Therefore, lignin deposition was analyzed in *Arabidopsis* stems to determine if lignin is involved in plant response to cesium stress through syringic acid (Figure 4). Soil-grown *Arabidopsis* stems were cryosectioned and stained for syringyl lignin after treatment with benzoic acid, sinapyl alcohol, cesium chloride, syringic acid, or a combined treatment of cesium and syringic acid. Benzoic acid is not known to be associated with lignin synthesis, and this was confirmed in the present results as the level of lignin staining in benzoic acid-treated stem sections was similar to that of the water-treated control (Figure 4). Treatment of plants with the syringyl lignin precursor, sinapyl alcohol, resulted in increased lignin deposition in interfascicular fibers (Figure 4). Interestingly, the cesium and syringic acid treatments also resulted in increased lignin deposition in interfascicular fibers, similar to the sinapyl alcohol treatment. No additional lignin deposition was observed, however, in the sections from plants that received a combined cesium and syringic acid treatment (Figure 4). These findings suggest that lignin may play a role in regulating plant response to cesium stress. Additionally, syringic acid also appears to be a positive mediator between lignin and cesium stress resulting in an increase in cesium tolerance.

### 2.4. Absence of REF4 Expression Negates Syringic Acid Alleviation of Cesium-Induced Growth Retardation

**Figure 5:**
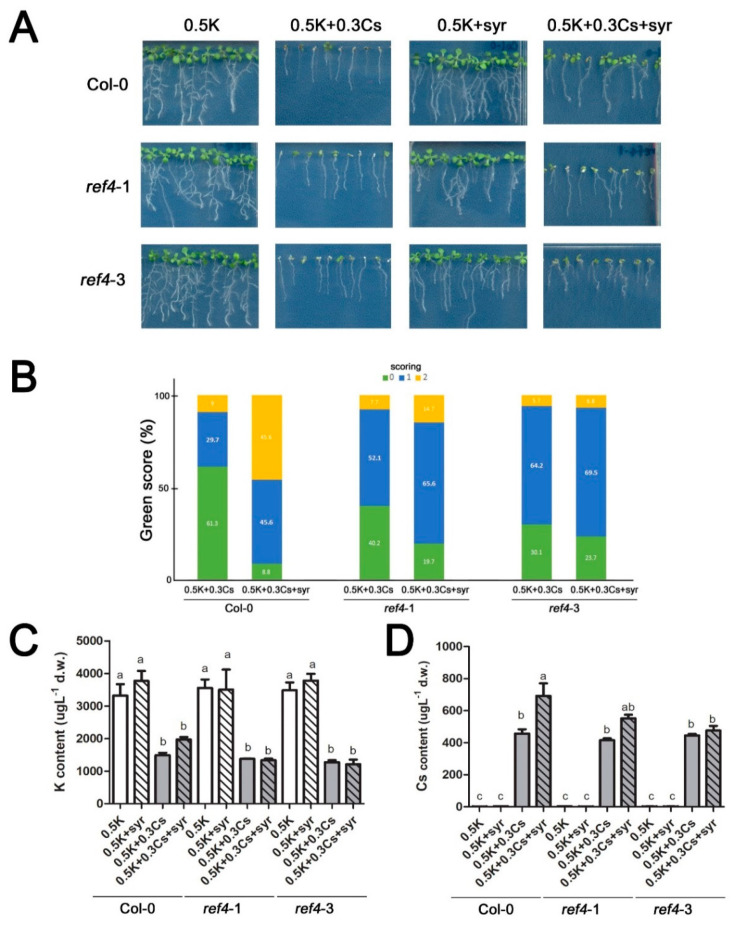
The absence of *REF4* expression negates the effect of syringic acid on the alleviation of cesium-induced growth retardation. (**A**) Two *Arabidopsis REF4* mutants (*ref4*-1 and *ref4*-3) were grown on media containing 0.5 mM potassium (K) and 0.3 mM cesium (Cs) with or without the addition of 100 μM syringic acid (syr). (**B**) Percentile green scores (left axis and numbers inside of bars) for *ref4* mutants and Col-0 treated with 0.5 mM K and 0.3 mM Cs with or without the addition of 100 μM syringic acid (syr) (*n* > 72). All of the seedlings used for the green scoring were also analyzed for potassium (**C**) and cesium (**D**) quantification. Statistically significant differences between treatment groups were determined by a one-way ANOVA with Bonferroni’s multiple comparison post-test and are indicated with different letters (*p* < 0.05). Error bars represent the SE.

**Figure 6:**
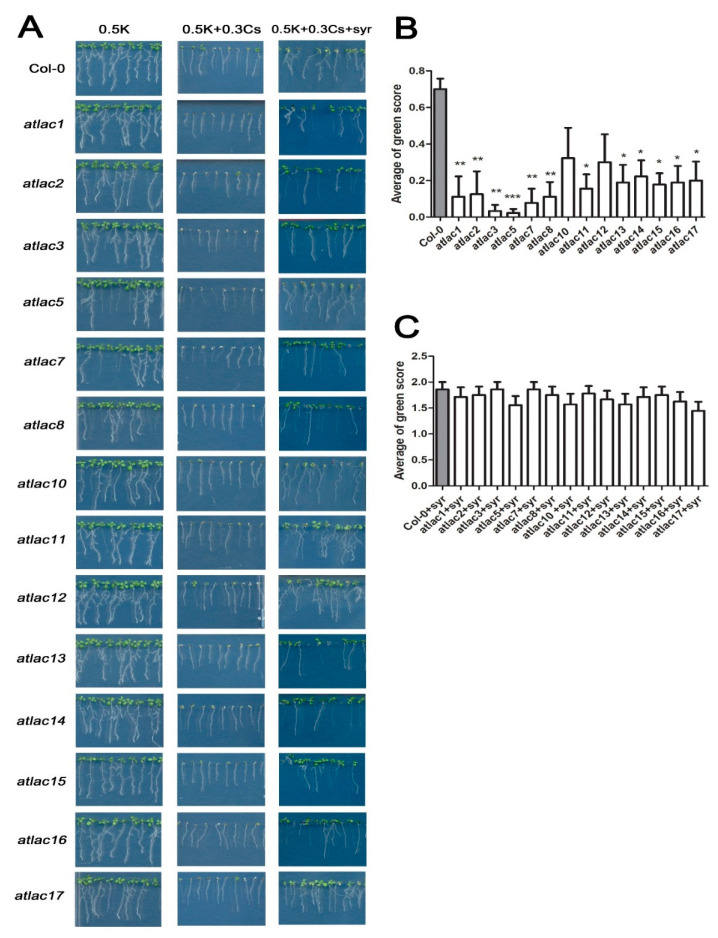
Response of *Arabidopsis* laccase mutants (*atlacs*) to cesium treatment. (**A**) A series of *atlacs* were grown on media containing 0.5 mM potassium (K) and 0.3 mM cesium (Cs) with or without the addition of 100 μM syringic acid (syr). Average green score for *atlac* mutants and Col-0 wild-type plants after 8 days of growth on media containing 0.3 mM cesium (**B**) and 0.3 mM cesium and syringic acid (**C**). Statistically significant differences between the mutant lines compared to control plants (Col-0) were determined by a Kruskal–Wallis test with Dunn’s multiple comparison post-test (* for *p* < 0.05, ** for *p* < 0.01, *** for *p* < 0.001). Error bars represent the SE (*n* > 60).

Two lignin defective mutants, *ref4*-1 and *ref4*-3 [28,29], and fourteen *Arabidopsis* laccase mutants, some of which are known to be involved in monolignol polymerization [41,42], were evaluated for their response to applications of cesium and syringic acid to better understand the role of lignin in plant response to cesium stress (Figure 5 and Figure 6). Aerial portions of cesium-treated *Arabidopsis* seedlings were given a score of 2 when they were green and fully expanded; a score of 0 when more than a half of the aerial portion of a seedling exhibited chlorosis; and a score of 1 when the appearance of the aerial portions was intermediate between a score of 2 and 0 [3]. The number of wild-type Col-0 *Arabidopsis* seedlings with a score of 0 was dramatically reduced from 61.3% to 8.8% as a result of the syringic acid treatment. Seedlings with a score of 2 also increased from 9.0% to 45.6% as a result of the syringic acid treatment (Figure 5B). Both *ref4*-1 and *ref4*-3 seedlings exhibited slightly improved tolerance to cesium stress relative to Col-0 wild-type plants when syringic acid was not applied (Figure 5). However, the number of *ref4*-1 and *ref4*-3 seedlings with a score of 0 was only reduced from 40.2% to 19.2% and 30.1% to 23.7%, respectively, by syringic acid treatment. Furthermore, the number of *ref4*-1 and *ref4*-3 seedlings with a score of 2 did not exhibit a large change as a result of the syringic acid treatment (Figure 5B). Interestingly, the levels of potassium and cesium were not altered in syringic acid-treated Col-0, *ref4*-1, and *ref4*-3 plants (Figure 5C,D). A slight increase in the level of cesium was observed again in Col-0 wild-type plants treated with syringic acid (Figure 5A).

Some of the laccases in *Arabidopsis* are known to be related to lignin synthesis through oxidative coupling reactions of radicals and key enzymes that catalyze the formation of monolignol. Reduced activity of laccases has been shown to reduce lignin content by 20~40% [41,42,43,44,45,46]. Therefore, we also evaluated *Arabidopsis* laccase mutants (*atlacs*) for their response to cesium stress and the application of syringic acid.

Mutants for 14 out of 17 *AtLAC* genes in *Arabidopsis* were evaluated for their response to cesium stress with or without the application of syringic acid to determine if *AtLACs* also play a role in syringic acid alleviation of cesium-induced growth retardation (Figure 6). Results indicated that all of the evaluated *atlacs*, with the exception of *atlac10* and *atlac12*, exhibited greater cesium-induced growth retardation relative to Col-0 wild-type plants (Figure 6B). However, no differences of green scoring were observed between Col-0 and *atlacs* mutants in their response to combined treatment with cesium and syringic acid (Figure 6C) indicating that the treatment with syringic acid was capable of alleviating the effect of cesium in the *atlacs* (Figure 6). Taken together, these findings suggest that lignin appears to be functionally involved in cesium-induced growth retardation and REF4 plays a role in the alleviation of cesium-induced growth inhibition by syringic acid.

### 2.5. Syringic Acid Has No Effect on Low Potassium Stress

**Figure 7:**
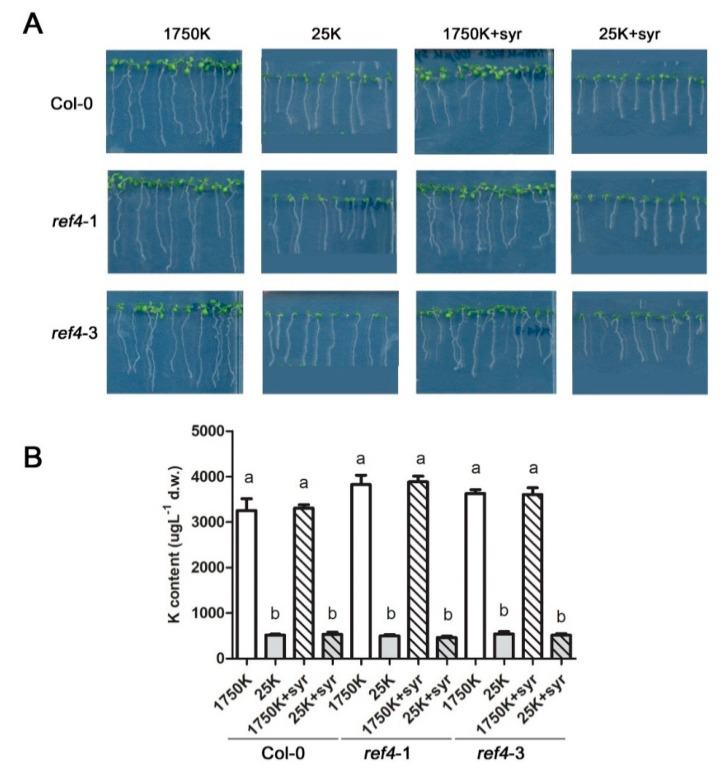
Syringic acid does not alter the response of *Arabidopsis* plants to low potassium. (**A**) *Arabidopsis* Col-0 wild-type and *ref4-1* and *ref4-3* mutant plants were grown on media containing 1750 μM or 25 μM potassium (K) with or without 100 μM syringic acid (syr) (*n* > 60). Each of the treated seedlings was used to quantify potassium (**B**) levels. Statistically significant differences between the treatment groups were determined by a one-way ANOVA with Bonferroni’s multiple comparison post-test and are indicated with different letters (*p* < 0.05). Error bars represent the SE.

The presence of cesium ions is known to compete with potassium uptake in plants and results in growth inhibition due to low potassium levels in cells [1,47]. To determine if syringic acid could attenuate potassium-starved growth defects, Col-0 plants, along with *ref4*-1 and *ref4*-3 mutants, were grown under low potassium conditions with or without syringic acid supplementation (Figure 7). Results indicated that the syringic acid did not alter the response of any of the evaluated *Arabidopsis* lines to low potassium conditions (Figure 7). Therefore, it appears that syringic acid specifically functions in alleviating cesium-induced growth retardation.

## 3. Discussion

Cesium is not considered to be beneficial to plants as it acts as a competitor for potassium uptake. The importance of understanding cesium transport and its effect in plants increased after the Fukushima nuclear power plant accident in Japan in 2011. Most studies, however, have focused on cesium-induced potassium deficiency and cesium transport in cells [7,48,49,50]. Potassium levels are regulated by phytohormones, such as jasmonic acid, cytokinins, auxin, ABA, and ethylene, as well as ROS [34,35,51,52,53,54]. Several studies have been conducted to determine if cesium transport or signaling is regulated by one or more of these factors. Among these factors, jasmonic acid biosynthesis and downstream signaling components were demonstrated to be induced by cesium, and the jasmonic acid-defective *Arabidopsis* mutants, *aos* and *coi1*-6, were shown to exhibit tolerance to cesium in roots, relative to wild-type control plants. Interestingly, the increased tolerance was not attributed to lower cesium accumulation or greater potassium accumulation [2]. A similar increase in tolerance was also observed in the present study when cesium-stressed wild-type Col-0 plants received a concurrent application of syringic acid, but this alleviation was predominantly observed in shoots (Figure 2). Jasmonic acid is involved in the phenylpropanoid pathway and lignin biosynthesis [55,56]. Cesium applications resulted in increased levels of jasmonic acid [1,2], syringic acid (Figure 1), and lignin deposition (Figure 4). Therefore, it is plausible that a triangular interaction among syringic acid, lignin, and jasmonic acid may play a role in cesium-induced growth inhibition.

Syringic acid is an abundant phenolic compound in a variety of plant species [13] and has many pharmacological properties, such as anticancer, anti-inflammatory, antioxidant, antiendotoxicity, hepatoprotection, neuroprotection, and antimicrobial activity [13,37,57,58,59,60,61,62]. Syringic acid is often found in natural soils at a range of 0.05~0.1 µmol·g^−1^ soil [63,64] and is a component of root exudates similar to other secondary metabolites [64,65,66,67]. Treatments with syringic acid have been reported to alter the composition of rhizosphere microbial communities [64], and it is also a product of lignin degradation [68]. In the present study, treatments with cesium resulted in an increase in syringic acid, specifically in shoots (Figure 1). This response may be related to the increased synthesis of phenylalanine, a major component in the phenylpropanoid pathway, and in response to cesium stress that only occurs in shoots and not in roots [9]. Unlike the plant response to potassium starvation, cesium stress induces several defects in aerial portions of plants, including growth inhibition and leaf chlorosis [1,3,9,47,69], and syringic acid also did not attenuate any potassium deficient responses (Figure 7). Specifically, treatment of *Arabidopsis* growing on media containing cesium with syringic acid resulted in a significant reduction in cesium-induced growth retardation (Figure 2A) without altering potassium levels (Figure 2B). Instead, an increase in cesium accumulation was most likely due to the increase in cell vigor (Figure 2C). Syringic acid confers structural integrity to lignin and is released during the degradation of lignin by fungal laccases [13,23]. As shown in Figure 3, the application of syringic acid resulted in greater lignin deposition, while cesium also increased lignin deposition in interfascicular fibers. This finding suggests that lignin synthesis is a component of plant response to cesium stress and that syringic acid may represent a link between lignin deposition and cesium stress in plants. REF4, a subunit of a large transcriptional coregulator, regulates lignin biosynthesis, and plant laccases play a role in lignin polymerization [42,70], as well as in the oxidation of syringic acid [46]. *Arabidopsis ref4* mutants, and some of the *atlac* mutants, have been shown to exhibit reduced lignin deposition in xylem elements [28,29,42,70]. The *ref4* mutants were slightly tolerant to cesium stress (Figure 5) and some of the *atlac* mutants were more sensitive to cesium stress (Figure 6), a finding that also indicates that lignin plays a role in plant response to cesium stress. Interestingly, the alleviation of a cesium-induced growth defect by syringic acid did not occur in the *ref4* mutants (Figure 5) but was observed in the *atlac* mutants, similar to Col-0 wild-type plants (Figure 6). Therefore, we hypothesized that the lignin deposition levels may not be the direct contributing factor responsible for alleviating the cesium-induced growth defect by syringic acid. Syringic acid-mediated alleviation of cesium stress may not be determined by lignin biosynthesis or degradation. Instead, it is possible that it may be controlled by more upstream components of the lignin biosynthesis pathway, such as regulators of phenylpropanoid homeostasis or by crosstalk with phytohormone pathways.

A lignin precursor, sinapyl alcohol, only exhibited a slight alleviation of cesium-induced growth retardation, which differed from the effect of syringic acid. In contrast, benzoic acid and its derived phytohormone, salicylic acid, did not have any effect on cesium response (Figure 3). In the case of treatment with DMBQ, it attenuated the growth inhibition by cesium at a mild level. DMBQ provokes calcium signaling in roots and defense-related gene expression [71]. Syringic acid has a greater effect in managing the response to cesium-induced growth defects in shoots versus roots, whereas DMBQ is known to play a role in roots [71]. Collectively, these findings indicated that cesium signaling is functionally involved within some parts of the phenylpropanoid pathway. Additionally, one of the final products of the pathway, lignin, may also be affected by cesium response via the transcriptional regulatory components of lignin biosynthesis, including REF4. REF4 has been suggested to be required for the homeostatic balance of the phenylpropanoid pathway [28], and semidominant mutations of *REF4* were shown to lead to an overall reduction of phenylpropanoid pathway compounds [28,31]. It is plausible that the balance of the phenylpropanoid pathway that is regulated by syringic acid and REF4 may be a critical regulatory factor for cesium response in plants. Additional characterization of the phenylpropanoid pathway, including an REF partner, MED5b, and its complex, will be carried out to further elucidate their functional roles in plant response to cesium.

In the present study, we provide the first demonstration of a link between cesium response and lignin in plants through the involvement of syringic acid. Our findings suggest that syringic acid and the REF4-mediated phenylpropanoid pathway play a certain role in the regulation of plant response to cesium, including cesium-induced growth inhibition. The regulation of cesium response, however, may not be due to lignin biosynthesis but rather by upstream regulatory components associated with lignin biosynthesis, such as *REF4* and jasmonic acid. A more detailed elucidation of the regulatory components responsible for syringic acid alleviation of cesium-induced growth inhibition will be performed in future research studies.

## 4. Materials and Methods

### 4.1. Plant Material and Growth Condition

Wild-type control *Arabidopsis thaliana* L. (Heynh) ecotype Columbia-0 (Col-0) and various lignin- and hormone-related mutants were used in this study. The mutant seeds of *atrboh*-c, *atrboh*-g, *atrboh*-h, and *atrboh*-i were obtained from Dr. Elizabeth Vierling (University of Massachusetts Amherst, Amherst, MA, USA) [39]. The mutant seeds for *ref4*-1 (CS66581), *ref4*-3 (CS66582), *atlac1* (CS482637), *atlac2* (Salk_010994C), *atlac3* (Salk_031901C), *atlac5* (Salk_092440C), *atlac7* (Salk_003265C), *atlac8* (Salk_082400C), *atlac10* (Salk_083241C), *atlac11* (Salk_063746C), *atlac12* (Salk_125379C), *atlac13* (Salk_052328C), *atlac14* (Salk_011767C), *atlac15* (CS2105589), *atlac16* (Salk_064093C), and *atlac17* (Salk_016748) [45] were obtained from the Arabidopsis Biological Resource Center (ABRC) (https://abrc.osu.edu/). Previously obtained *ein2*-1 [51], *ipt1,3,5,7*, and *ahk2*;3 [53] seeds were also used in this study. The seeds of all plant lines were surface sterilized with 70% (*v*/*v*) ethanol and 0.05% (*v*/*v*) Triton X-100, rinsed in Milli-Q water, and subsequently placed on media containing 0.5 mM KCl, 50 μM H_3_BO_3_, 10 μM MnCl_2_, 2 μM ZnSO_4_, 1.5 μM CuSO_4_, 0.075 μM NH_4_Mo_7_O_24_, 74 μM Fe-EDTA, 0.5 mM phosphoric acid, 2 mM Ca(NO_3_)_2_, and 0.75 mM MgSO_4_ at pH 5.8 with Ca(OH)_2_, 1% (*w*/*v*) sucrose, and 1% (*w*/*v*) SeaKem LE Agarose (Lonza, Basel, Switzerland). Media supplemented with 300 μM CsCl was used to administer cesium stress and media containing 25 μM KCl instead of 0.5 mM KCl was used to establish a potassium deficiency condition [3]. Medias supplemented with either 100 μM syringic acid, 100 μM sinapyl alcohol, 10 μM 2,6-dimethoxybenzoquinone (DMBQ), 10 μM benzoic acid, or 10 μM salicylic acid were also used. After stratification for 3 days at 4 °C in the dark, seeds were germinated and grown on vertically positioned plates in a controlled growth cabinet for 8 days, under a 16/8 h light/dark cycle (80 to 100 μmol m^−2^ s^−1^) at 22 °C.

### 4.2. Elemental Analysis

At least three biological replicates, consisting of 60–72 pooled seedlings in each replicate, were analyzed for each treatment group. Whole eight-day-old plantlets were harvested, rinsed in Milli-Q water, and dried in an oven at 65 °C for 3~4 days. Two ± 0.1 mg samples of dried seedlings were extracted in 1 mL of 60% (*v*/*v*) HNO_3_ by heating at 125 °C for 1 h. The resulting solutions were then diluted with Milli-Q water to 10 mL. Cesium and potassium levels were measured by an AAnalyst 200 flame atomic absorption spectrometer (PerkinElmer) or an inductively coupled plasma mass spectrometry (NexION^®^ 300 ICP-MS System, PerkinElmer, Waltham, MA, USA). The concentrations of the elements were calculated against a standard curve constructed for each element [72]. Statistically significant differences between treatment groups or plant lines were evaluated with a one-way ANOVA with Bonferroni’s multiple comparison post-test using GraphPad Prism software (version 5, GraphPad Software, La Jolla, CA, USA).

### 4.3. Phenotyping

Aerial portions of cesium-treated *Arabidopsis* seedlings were scored as previously described [3]. At least seven plates and more than 72 seedlings per each condition were used for analysis. The plates were prepared as described above. Briefly, all of the tested *Arabidopsis* seedlings with fully expanded, green aerial portions of stems and leaves were given a score of 2, plants with more than half the number of leaves exhibiting chlorosis were given a score of 0, and intermediate phenotypes (less than 50% seedlings showed chlorosis) were given a score of 1. Green score was analyzed with Kruskal–Wallis test with Dunn’s multiple comparison post-test using GraphPad Prism software.

### 4.4. Syringic Acid Quantification

Syringic acid was extracted using a modified procedure developed for metabolic profiling of cell walls in *Arabidopsis* tissues [73]. *Arabidopsis* tissues, 50 mg DW of shoot and 12.5 mg DW of root, were extracted in 1 mL of extraction solvents: 50% (*v*/*v*) MeOH, two parts MeOH, H_2_O, 0.5% (*w*/*v*) SDS, 1 M NaCl, MeOH, acetone, and n-hexene. The extraction was carried out for 15 min on a rotary shaker and each extract solvent was then centrifuged at 17,800× *g* for 15 min, and the supernatants were removed. Precipitation was carried out using a centrifugal evaporator for 2 h (SpeedVac SPD2010, Thermo Fisher, Pittsburgh, PA, USA). The precipitates (10 mg DW of shoot and 5 mg DW of root) were then further extracted in 3 mL of NaOH for 15 min on a rotary shaker and the solvent was subsequently replaced with nitrogen gas. After heating at 80 °C for 24 h in a dark room, 3 mL of HCl and 6 mL of ethyl acetate were added to the extract. After vortexing, 200 μL upper layer (organic layer) of the extraction solvent were collected and dried using a centrifugal evaporator for 2 h. The extracts were resolved with 100 μL of 80% (*v*/*v*) MeOH containing an internal standard (33.6 nM of lidocaine) and then filtered through a 0.2 µm pore size centrifugal filter (Ultrafree-MC Centrifugal Filter, 0.2 µm pore size, hydrophilic PTFE, 0.4 mL volume, non-sterile, Merck, Darmstadt, Germany). Using a liquid handling system (MicrolabSTARplus, Hamilton Company, Reno, NV, USA), 10 μL of filtered solvents were dried up by N_2_ gas, and resolved in 250 μL of LC–MS grade pure water (FUJIFILM Wako Pure Chemical Corporation, Osaka, Japan), and filtered by a 384-well filter (MultiScreenHTS 384 well, Merck, Kenilworth, NJ, USA). One μL of the solvents was used for liquid chromatography with tandem mass spectrometry analysis. A syringic acid standard was purchased from Sigma-Aldrich (St. Louis, MO, USA), and detection conditions were optimized for LC-QqQ-MS (UPLC-TQS, Waters, MA, USA). LC conditions were as previously reported [74]. One μL of extracted solvent was injected into the LC-QqQ-MS device, and peak area values obtained in the LC–MS/MS analysis were calculated using MassLynx software (version 4.1, Waters, MA, USA). The peak area values were used to plot the bar graphs and a *t*-test was performed to determine the statistical significance using GraphPad Prism software.

### 4.5. Lignin Staining

Histochemical localization of lignin in *Arabidopsis* stems was performed using the Maüle staining method [75,76,77]. Stems of soil-grown bolted *Arabidopsis* plants that had been treated for 3 consecutive days with 10 mL each of either Milli-Q (control), 10 mM cesium, and/or phenolic compounds (1 mM syringic acid, 1 mM sinapyl alcohol, or 1 mM benzoic acid) were soaked in water under a vacuum and then frozen. Cryosections (20 µm thick) of stem cross-sections were obtained with a Leica Cryo-microtome CM3050S (Leica, Wetzlar, Germany) at −30 °C. The sectioned stem samples were moved to glass slides, covered with coverslips, and thawed. Sections were then stained with 1% (*w*/*v*) KMnO_4_ for 2 min, followed by rinsing with Milli-Q three times. Washed sections were then destained with 10% (*w*/*v*) HCl for 2 min and subsequently rinsed three times with Milli-Q water at room temperature. Samples were then immersed in concentrated NH_4_OH, and digital photomicrographs were taken on an Olympus BX51 microscope equipped with a digital camera (Olympus cellSens and DP26, Tokyo, Japan).

## Supplementary Materials

Supplementary Materials can be found at https://www.mdpi.com/1422-0067/21/23/9116/s1.

## Abbreviations

AtLAC	*Arabidopsis* LACCASE
DMBQ	1,6-dimethoxybenzoquinone
NADPH oxidase	nicotinamide adenine dinucleotide phosphate oxidase
REF4	REDUCED EPIDERMAL FLUORESCENE 4
