# Supplementary material for: Syringic Acid Alleviates Cesium-Induced Growth Defect in Arabidopsis
Source: Int J Mol Sci. 2020 Nov 30;21(23):9116. doi: 10.3390/ijms21239116 (PMC7730055; doi:10.3390/ijms21239116)
Supplement: Supplementary file 1 [file ijms-21-09116-s001.pdf]

Supplementary Materials

Figure S1

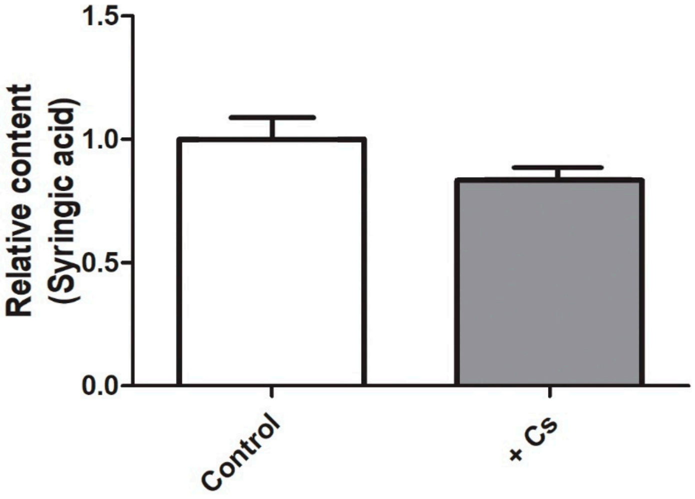

Figure S1. Relative levels of syringic acid in *Arabidopsis*. The level of syringic acid was analyzed in roots of cesium-treated *Arabidopsis* plants by liquid chromatography coupled with tandem quadrupole mass spectrometry. Results are presented as a ratio between the levels of syringic acid in untreated control plants versus the levels in cesium-treated plants. The white bar indicates the level of syringic acid in roots of control plants and the grey bar indicates the level of syringic acid in roots of cesium-treated *Arabidopsis* plants, and error bars represent the standard error (SE). A *t*-test was conducted and no significant difference between treated and untreated plants was observed ($n = 4$).

Figure S2

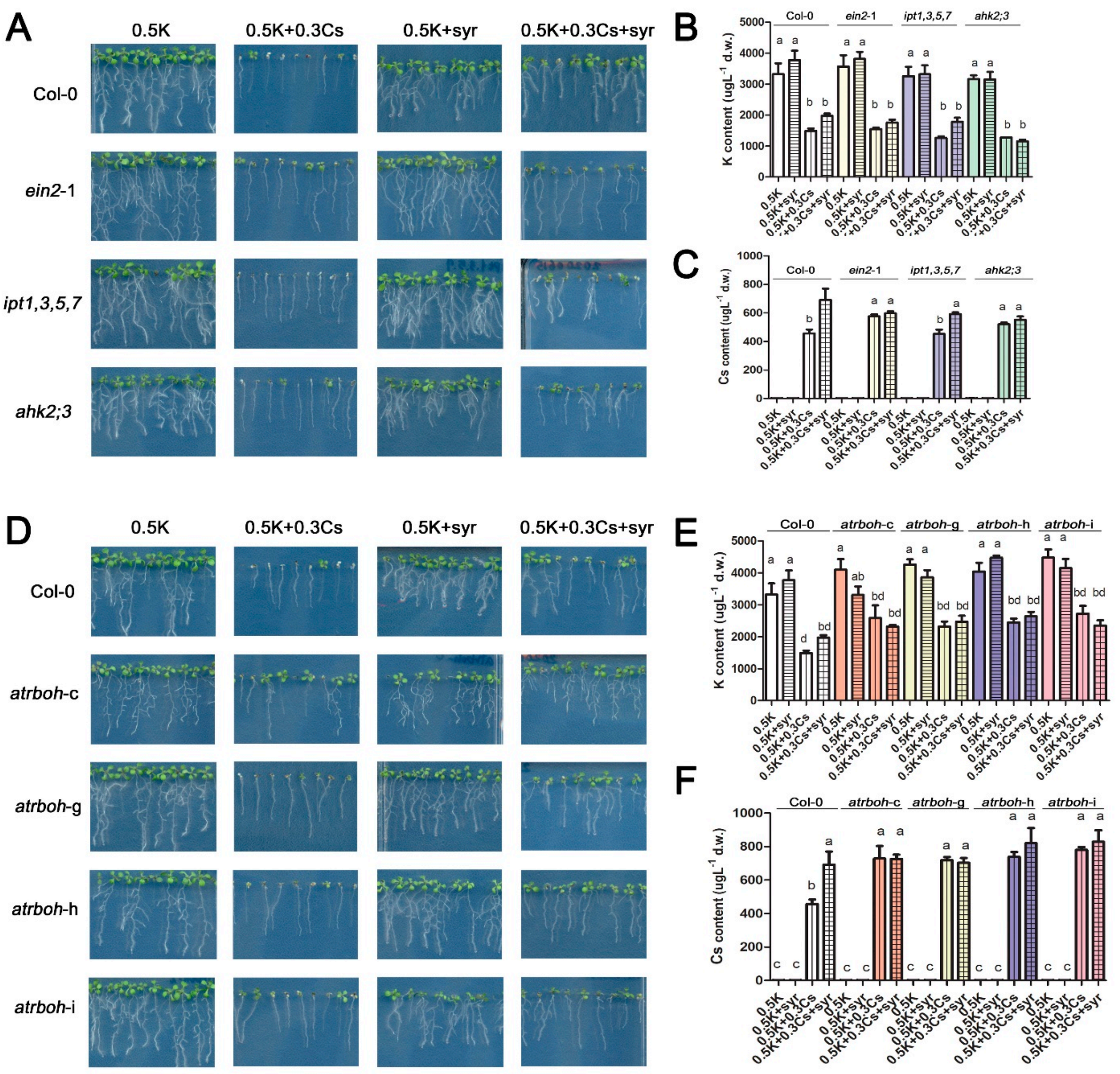

Figure S2. Response of ethylene and cytokinin mutants (**A**) and NAPDH oxidase mutants (**D**) to cesium and syringic acid. *Arabidopsis* mutants were grown on media containing 0.5 mM potassium (**K**) and 0.3 mM cesium (Cs) with or without 100 μM syringic acid (syr) for 8 days. Seedlings were analyzed for potassium (**B**,**E**) and cesium (**C**,**F**) concentrations. Statistically significant differences between treatment groups were determined by a one-way ANOVA with Bonferroni's multiple comparison post-test and indicated with different letters ($p < 0.05$). Error bars represent the SE.
